# Correspondence

**Published:** 1881-06

**Authors:** 


### CORRESPONDENCE.

*Editor Dental Register:—*

Herewith I send an extract from a paper read before the Illinois State Dental Society, in which I endeavor to present a few thoughts on the use of materials for filling teeth, and the care of the teeth after this operation.

Those who follow Dr. Arthur in the same care taken by him in his operations, require courage and indomitable energy. Those operators who are constantly beveling teeth for self-cleansing surfaces, necessarily avoid contour operations. Young operators who produce frequent contour fillings, must occasionally fail; and if they do not protect cusps of pulpless teeth, some must be lost, and need we be surprised that from these the "new departure" receives new allies.

Young graduates sometimes *risk* their reputation on amalgam, and afterwards wished they hadn't.

An old practitioner may use cement and gutta-percha with a hope of saving his aching back and enervated nerve system, while, theoretically, he expects as good fillings as before—but, alas, this proves the theory a practical untruth!

Therefore let us cull the principles which, when carried out will result in durable operations, remembering as well, that we must necessarily, occasionally fail.

When gold, amalgam, tin and cement, remain in teeth only a few weeks, the retaining grooves are usually at fault. When they remain a year or more, we must look further for the true cause of failure.

As we depend largely on the pits, grooves and general anchorages to retain our finished fillings, let us look at their preparation a few minutes. The rubber dam should be applied where practical, before commencing to excavate, for there are few cavities which can be prepared properly without its previous careful adjustment. Grooves near the grinding surfaces of teeth should be strong, and made in a direction to accommodate the occluding force of the antagonizing tooth, so that the effect of grinding the teeth in masticating food, will be to concentrate the force toward the center of the organ, rather than distributing to the periphery. Also suppose it requires two hundred grindings of the teeth at each meal to prepare food for insalivation, at this rate over two hundred thousand occlusions will be required each year, and suggests the strength requisite in the grooves to overcome this force. Pits or grooves in large cavities in proximal surfaces of Incisors and Cuspids, if made wholly in the dentine commencing near the cutting edge, and proceeding towards the pulp, being careful not to approach too near that organ, will retain a filling better and be more easily filled, than when made in the opposite direction. In this way the point of the tooth is not weakened and the cutting force of the lower teeth will not be so apt to dislodge the filling if these retainers are well filled. This pit or groove should not be filled with soft foil, but cohesive. Very narrow, short strips of, say No. 20 or 30, are best, filling very carefully, overlapping the edges in order to weld the gold in the retainer, by bands, to the body of the filling. A small gold screw, if driven into the dentine at the point, is still better allowing the screw to cut its own thread. This is a difficult operation, for, if the sides of the screw touch the enamel plates they will be checked, and after filling, either discoloration or decay ensues. In large cavities in bicuspids and molars, the retainers should be made in the dentine if possible, using the enamel to assist in retaining the filling only when it is very resistant, and only then to assist in forming a general dovetail. There are but few exceptions to this rule. When making general anchorages in grinding surfaces, remember that all cusps in pulpless teeth are to be protected from being driven asunder. Retaining pits in the cervical portion of a cavity should be wholly in the dentine.

Fillings are lost from cavities prepared by good operators, for, man in his entirety is imperfect, and his labor partaking of this bias must bear the imprint. Also the best patrons do not carry out strictly our simplest instructions for the preservation of their teeth. With this in mind we may examine the reasons of fillings being lost.

Tin fillings gradually wear away except on unused surfaces and usually leave the walls uninjured, if the cavities are properly prepared and carefully filled. Gutta-percha is worn away as much as tin ; but it does not preserve the walls as well, for sometimes we see extensive decay at the cervical margins of these fillings. Similar decay occurs around cement fillings when we have been careful to avoid it, and even in those teeth that are said to indicate its use, chalky and pulpless ones. These cavities are due either to minute air vesicles at the margins, improperly mixing, improper proportions, slowness in inserting the filling, or in not keeping the filling dry long enough. In cavities in chalky teeth in surfaces cleansed in masticating, and in proximal where they do not extend to the gingival border, cement is the best filling until a change of the tooth structure takes place. In healthy mouths, where nothing is eaten between meals, we may succeed with cement in cavities extending under the gum. Decay may occur anywhere around an amalgam filling, or disappoint by preserving the tooth. In my observations these are rules and not exceptions. A good cement wears away less rapidly than tin, and often leaves the walls in good condition, while amalgam does not wear away except at the margins. Cement is easy of insertion and disappoints by cavities forming under the gum nearly exposing the pulp, while the surface seen looks well. Amalgam may have decay under its entire surface and the pulp slowly perishing, while its external surface may be bright, and nothing but a mere line of demarkation appear as a sign of this destruction below. Cement may be inserted with some assurance of knowing the issue, but with amalgam, no matter how carefully one follows instructions, or supposed rules founded on experience, failure is as likely as success.

These defects are rarely noticeable in gold fillings, bearing in mind painstaking and patiently-executed operations.

J. M. Hurtt.

*Peoria, May* 20, 1881.

Xenia, O., May 21st, 1881,

*Editor Dental Register*,

My Dear Sir:—

If you will allow me to begrudge you as to any of your privileges, let it be, that you can go when and where you please, and consequently, visit the various meetings of the dental societies, see our professional brethren, rub against them, and thus get the rust and the mold worn off yourself, so as to fit you for fresh duty in your office, and, especially, for your journalistic work, thus getting fresh items for your editorials and articles of merit for your original communication department—think of it, while I am caged in my "sanctum"—or *sanitarium*, perhaps is better—seeing almost no one, grinding out my specials; too often, I fear, from the dead past, wondering how you all look, and feeling almost hopeless as to being ever able to see how you do look in a societary capacity. W-h-e-w! What a long sentence! but then it is a w(h)ail, and ought to be large.

It would have given me extreme pleasure to have been with you at Rock Island and Davenport; but then, I must learn "in whatsoever state I am therewith to be content." I was weak enough to fancy that I had learned this lesson—had made the attainment so desirable—but not getting to the March meetings, in Cincinnati, showed me how much I have yet to learn; for, I am ashamed to tell you, I was as unresigned as a spoiled child. But, after all, is not that consistent, for what am I but a spoiled, petted child, though no longer a young one? Spoiled and petted by my professional brethren, by my friends in general, yourself not excepted; humored and caressed by my Father in Heaven.

But you got to the meetings, and as the Register is, therefore, likely to be enriched, we shall read, and enjoy, and possibly make a friendly draw on it for the pages of the journal.

In the mean time, I may as well tell you, for it will get out; we expect, after June, to issue the journal the first, and not the middle of the month, as heretofore. So, if you and your readers shall conclude to furnish us contributions, as in the natural kindness of your hearts you will be sure to do, let us have them by the 10th of the month preceding.

Editorial labor seems quite natural; the only difficulty I find is that I often write "*Register*," when I mean JOURNAL. But they say a fellow has a fancy for the name of his first girl, even after she has jilted him; but I know nothing about that, for the Register never jilted me, and I regard her as a good old girl yet.

I am thinking of the July and August meetings, as the small boy thinks of the cakes and candies on the upper shelf of his mother's cupboard, quite out of his reach. Yet I sometimes hope the American Dental Association may be brought within reach of me once more, say Cincinnati, Indianapolis, Detroit—then if a little stronger than common, I could attend. You may rest assured I am not content to be absent, if possible to be present.

At last it is done—that is, it's been and gone and got done, viz: the recognition of "Dentistry" as a specialty in medicine. Of course everyone must know that dental science is a part, or specialty, in medical science; but is the dental profession composed of pratitioners of a medical specialty? is not quite the same question. At all events, the late meeting of the American Medical Association, made provision for a Section on Dentistry. Some objected to the form of expression, wanting "Dentology," "Dental and Oral Surgery," or something else substituted for "Dentistry;" but Dentistry it is, and Amen! So let it be!

The objection, with some is that it takes in Mechanical Dentistry, with its plaster, plates, and appliances, but let it. Surgery takes in its knives, splints, and pulleys; and so of other sections, they take in their traps and baggage, whatever they have; and if mechanical dentistry is too low to be taken in it must be elevated. We like the term "Dentistry." "They used to call a spade a spade."

But I must weary you no further. I am not responsible for the literary character of the Register; but if you disfigure the pages with this stuff, you'll want revenge. So, maybe you'll send me something for the *Journal* "just for spite." Send it along. Who's afraid?

And now, with best wishes for you and the Register, I am, with the compliments of the *Journal*, your friend,

GEO. WATT.

---

*Editor Register.*

DEAR SIR: Let us be thankful, for have we not been recognized? Yea verily.

The American Medical Association at its last annual meeting held in May, at Richmond, Va., decided to add unto itself a section on dentistry. To what end this has been done is not fully explained but probably to the tail end. This will have the effect of making the tail a little longer, and enable it to take a broader sweep in its attack upon flies, at any rate the profession can furnish wind enough to make their sessions longer if not everlasting.

It has been said that this meeting of the association was one of the most unprofitable ever held. Now this may be accounted for from the fact that after the tail was spliced primary adhesion failed to take place, inflammation set in and induced such a degree of constitutional disturbance that the usefulness of the whole body became impaired.

What this dental appendage is expected to accomplish we do not know; it makes no difference, it is sufficient to know that we have been recognized or medicated as it were, and can now lay claim as a medical brother. It will moreover have a tendency to sooth those troubled souls who have been fearful lest they should be considered as little above the dignity of the blacksmith and who have been clamoring loud and long to be placed under the ægis of the medical profession. For their sake we hope the medical association will not find it necessary to retrogress.

This generous and magnanimous act of recognition on the part of the medical association should be rewarded. So let the Dental Association when it meets in New York, return the compliment by creating a medical section and give the doctors a chance.

M. D. D. D. S.

